# Beat-to-beat alterations of acoustic intensity and frequency at the maximum power of heart sounds are associated with NT-proBNP levels

**DOI:** 10.3389/fcvm.2024.1372543

**Published:** 2024-04-02

**Authors:** Kazuhiro Fujiyoshi, Minako Yamaoka-Tojo, Kanako Fujiyoshi, Takumi Komatsu, Jun Oikawa, Kunio Kashino, Hitonobu Tomoike, Junya Ako

**Affiliations:** ^1^ Department of Cardiovascular Medicine, Kitasato University School of Medicine, Sagamihara, Japan; ^2^ Department of Rehabilitation, Kitasato University School of Allied Health Sciences, Sagamihara, Japan; ^3^ Department of Functional Restoration Science, Kitasato University Graduate School of Medical Sciences, Sagamihara, Japan; ^4^ Department of Kitasato Clinical Research Center, Kitasato University School of Medicine, Sagamihara, Japan; ^5^ Bio-Medical Informatics Research Center, NTT Basic Research Laboratories, Atsugi, Japan

**Keywords:** auscultation, cardiovascular disease, heart failure, body mass index, heart sound

## Abstract

**Background:**

Auscultatory features of heart sounds (HS) in patients with heart failure (HF) have been studied intensively. Recent developments in digital and electrical devices for auscultation provided easy listening chances to recognize peculiar sounds related to diastolic HS such as S_3_ or S_4_. This study aimed to quantitatively assess HS by acoustic measures of intensity (dB) and audio frequency (Hz).

**Methods:**

Forty consecutive patients aged between 46 and 87 years (mean age, 74 years) with chronic cardiovascular disease (CVD) were enrolled in the present study after providing written informed consent during their visits to the Kitasato University Outpatient Clinic. HS were recorded at the fourth intercostal space along the left sternal border using a highly sensitive digital device. Two consecutive heartbeats were quantified on sound intensity (dB) and audio frequency (Hz) at the peak power of each spectrogram of S_1_–S_4_ using audio editing and recording application software. The participants were classified into three groups, namely, the absence of HF (*n* = 27), HF (*n* = 8), and high-risk HF (*n* = 5), based on the levels of NT-proBNP < 300, ≥300, and ≥900 pg/ml, respectively, and also the levels of ejection fraction (EF), such as preserved EF (*n* = 22), mildly reduced EF (*n* = 12), and reduced EF (*n* = 6).

**Results:**

The intensities of four components of HS (S_1_–S_4_) decreased linearly (*p* < 0.02–0.001) with levels of body mass index (BMI) (range, 16.2–33.0 kg/m^2^). Differences in S_1_ intensity (ΔS_1_) and its frequency (Δ*fS_1_*) between two consecutive beats were non-audible level and were larger in patients with HF than those in patients without HF (ΔS_1_, *r* = 0.356, *p* = 0.024; Δ*fS_1_*, *r* = 0.356, *p* = 0.024). The cutoff values of ΔS_1_ and Δ*fS_1_* for discriminating the presence of high-risk HF were 4.0 dB and 5.0 Hz, respectively.

**Conclusions:**

Despite significant attenuations of all four components of HS by BMI, beat-to-beat alterations of both intensity and frequency of S_1_ were associated with the severity of HF. Acoustic quantification of HS enabled analyses of sounds below the audible level, suggesting that sound analysis might provide an early sign of HF.

## 1 Introduction

Elevated jugular venous pressure and the presence of a third heart sound precede the onset of heart failure (HF) symptoms by days to months (1). To prevent the clinical worsening of cardiovascular disease (CVD), early identification of HF becomes crucial. There is no doubt that physical examination raises suspicion for HF. Therefore, diastolic heart sounds (HS) such as third sound (S_3_) or fourth sound (S_4_) have garnered attention for their clinical significance in the presence of HF (2–4), and classical diagnostic tools have been modernized to assess the clinical significance of diastolic HS (5). However, Marcus et al. (2) found that the presence of an S_3_ had a sensitivity of 32%–52% and a specificity of 87%–92% and the presence of an S_4_ scored lower. When an implantable HS sensor was chronically embedded in animal models, the S_3_ intensity was able to detect elevated left atrial pressure, defined as >25 mmHg, with a sensitivity of 58% and specificity of 90% (6). Using the electro-stethoscope, physicians can recognize not only changes in audible HS but also the presence of S_3_ and/or S_4_ equivalent vibrations, which are inaudible to the human ear, during the protodiastolic (2, 3) and presystolic period (4, 7). To understand the acoustic features of HS, we introduced a digital acquisition and processing of HS to minimize subjective differences in audibility or visual recognition of phonocardiograms in patients with CVD. We expected that acoustic quantification of HS on intensity (dB) and frequency (Hz) would enable comparisons among patients with HF as well as with other clinical findings.

## 2 Methods

### 2.1 Study participants

The present study design was based on the Cardiovascular Second Prevention Center (CSPC) of the Kitasato University Hospital. Patients who were admitted to this hospital due to acute CVD sought care at the CSPC outpatient clinic to prevent CVD recurrences every other year of their own volition. From this hospital-based cohort in which stableness and chronicity of CVD were ascertained, a cross-sectional single-center exploratory study was designed to assess the clinical usefulness of quantitative HS analyses.

An adequate number of participants were pre-calculated in previous studies describing an association of HF in the general population and patients with CVD (8, 9). Six to eight patients with HF are minimally required for the statistical analysis; however, we expected that forty patients with CVD would be needed for the present analysis. The Kitasato University Hospital Research Ethics Committee approved the present study protocol (Clinical Trial Registration No. B22-060). Between 4 August 2023 and 6 October 2023, 40 consecutive outpatients, aged between 46 and 87 years with chronic CVD, were enrolled after receiving written informed consent from each patient. In the present participants, S_3_ and S_4_ were not audible at the outpatient clinic. The mean duration from their discharge of acute illness to the present CSPC visit was 8.9 ± 6.3 years.

### 2.2 Clinical evaluation and collecting HS

All participants underwent comprehensive clinical evaluations that included medical history updates, blood pressure measurements, electrocardiogram (ECG), echocardiography, chest x-ray, and blood tests on the same day. Blood collection for N-terminal pro-brain natriuretic peptide (NT-proBNP) was also performed during ordinary laboratory tests between 8:30 a.m. and 10:30 a.m. or 1:00 p.m. and 2:20 p.m.

**Figure 1:**
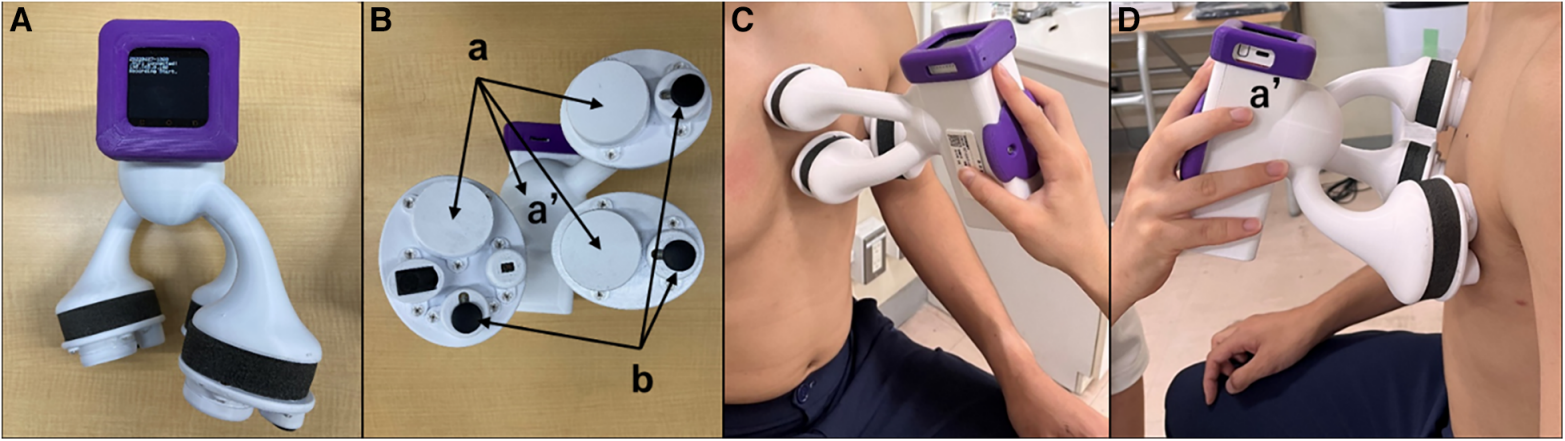
Depiction of handheld tri-antler chest piece to record heart sounds. Front view (**A**) of BMP-LD3 (handheld tri-antlers chest piece). The back side including four microphones, three of which are inside the chest pieces (a) and one in the connecting box. (b) Three electric terminals for ECG (**B**). Two microphones attached at the left upper and lower sternal border (**C**). One microphone attached at apex (**D**) The fourth microphone (a′) is in the housing (handle) to collect ambient noise used for noise cancellation purposes.

HS collection was performed on the same day or within 17.0 ± 8.1 days, in between a tight schedule of the regular health insurance services using the handheld tri-antler chest piece (BMP-LD3, NTT Basic Research Laboratories, Japan, Figure 1A). This device has four microphones. Each microphone is placed inside the chest piece in each antler to record HS, and the fourth one is inside the housing to record ambient sounds (Figure 1B). BMP-LD3 was designed to attach three chest pieces firmly and easily to the anterior chest wall at the sitting or supine position of the participant *per se*. When the device was attached to the anterior chest, two chest pieces were perpendicularly placed on the second intercostal space (heart base) and the fourth intercostal space, along the left sternal border (Figure 1C). The last third one was at the apex (Figure 1D). Each patient sat freely in the daily examination room. Sounds from four microphones were recorded for at least 10 s. HS collected through the handheld tri-antler chest piece (BMP-LD3) were saved digitally at a rate of 16 kHz in a memory inside the connecting housing of BMP-LD3 (Figure 1A) (10). The block diagram of the procedure for S_1_–S_4_ extractions and their analyses is presented in Supplementary Figure S1.

### 2.3 Analysis of HS

**Figure 2:**
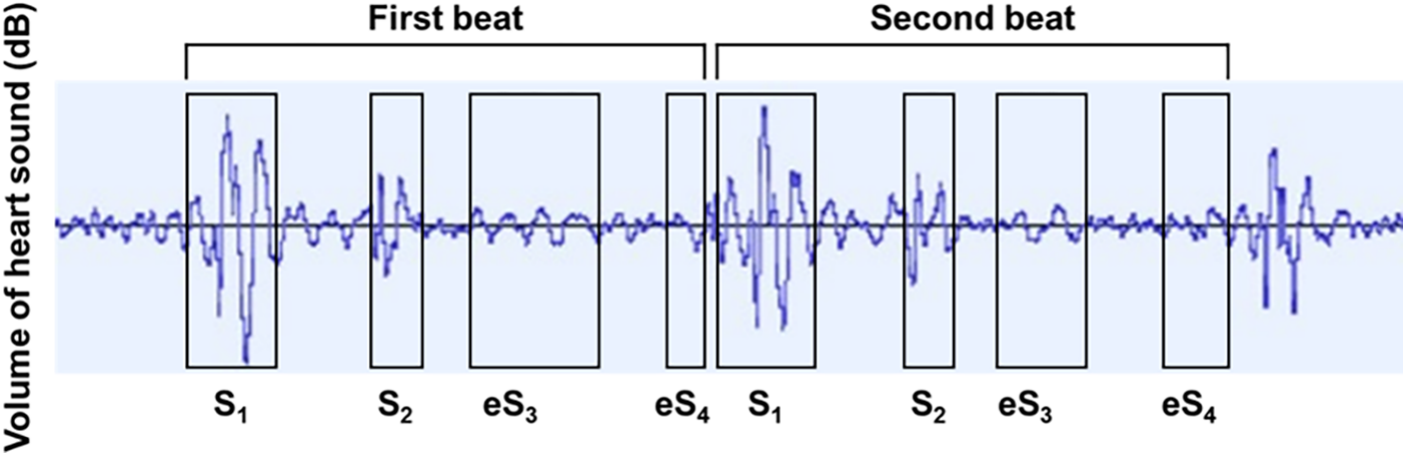
Phonocardiographic tracings in determining S_1_, S_2_, eS_3_, and eS_4_. S_1_, first heart sound; S_2_, second heart sound; eS_3_, equivalent to third heart sound; eS_4_, equivalent to the fourth heart sound.

Collected HS were analyzed by the audio editing and recording software (Audacity 3.3.3; Muse Group, Cyprus). Audacity, a sound recording software that is freely available, was used in the study for recording and analyzing HS (11, 12); signals captured through each chest piece were amplified by 30 dB, which was then simply subtracted by the level of the ambient sounds. Although the first (S_1_) and second sounds (S_2_) were easily recognized in all cases on the phonocardiogram, S_3_ and S_4_ were noted as tiny sound vibrations (Figure 2). To save S_3_ and S_4_ correctly, the signals at the fourth intercostal space chest piece along the left sternal border were chosen, and the other two signals at the base and apex chest pieces were used for identifying the timings of S_1_, S_2_, S_3_, and S_4_. We defined sound vibrations during protodiastole as S_3_ equivalent (eS_3_) and those during presystole as S_4_ equivalent (eS_4_). S_1_ is mostly constructed by three peaks from the baseline (13, 14). S_2_ is constructed by two peaks from the baseline (15, 16). From the end of S_2_ to the start of eS_3_, there is a sound-free pause as the early systolic baseline (17). Then, eS_3_ was defined as shown in Figure 2. eS_4_ is from the end of the mid-systolic baseline to the start of S_1_ (2, 4). Thus, the range length was different between the first beat and the second one.

We chose two consecutive beats, which were recorded with clear ECG and with the absence of respiratory noise. On these two beats, the average and their mathematical difference (Δ) of intensity (dB) and audio frequency (Hz) were taken for the analysis of the clinical characteristics of patients. The RR interval was measured by an ECG attached to the device. All measurements were performed by an independent clinical laboratory technologist who was blinded to the clinical features of each patient.

### 2.4 Definition of heart failure and clinical categories

HF and high-risk HF were defined as NT-proBNP ≥ 300 and ≥900 pg/ml, respectively (9). HF was also categorized on the levels of ejection fraction (EF). HF with reduced EF (HFrEF), mildly reduced EF (HFmrEF), and preserved EF (HFpEF) were defined as left ventricular ejection fraction (LVEF) <40%, 40%–50%, and ≥50%, respectively (18). Ischemic heart disease (IHD) included myocardial infarction, angina pectoris, and vasospastic angina. Atrial fibrillation (AF) was defined as absolute arrhythmia confirmed by ECG. The parameters of the echocardiogram follow the international guidelines for echocardiography (19). Valvular heart diseases were diagnosed by auscultation and echocardiography. The ECG wave graph and explanation of Q, R, and S waves at V1 and V5 of the ECG chest electrode are shown in Supplementary Figure S2. QRS duration was measured from the beginning of the Q wave to the end of the S wave (20). SV1 + RV5 voltage was defined as the sum of the S wave amplitude in V1 and the R wave amplitude in V5 (21). Cardiothoracic ratio (CTR) was defined as the ratio of the transverse dimension of the heart to the chest width measured on a chest radiograph of posteroanterior view (22).

### 2.5 Statistical analysis

Normally distributed continuous variables are expressed as mean ± standard deviation, whereas non-normally distributed values are expressed as median with interquartile range and were analyzed using Wilcoxon signed-rank test. Continuous variables were analyzed using unpaired *t*-tests. Categorical variables are reported as number (%) and were analyzed using the chi-squared test. Receiver operating characteristic (ROC) curves were constructed to assess the predicted occurrence of HF and high-risk HF and to determine the best cutoff of the sound intensity for detecting HF and high-risk HF. A multivariate regression analysis was performed to identify the factors associated with the presence of HF or high-risk HF among variables with values of *p *< 0.050 on the univariate logistic regression analysis. Statistical significance was defined as *p* < 0.050. JMP version 9.0 software (SAS Institute, Cary, NC, USA) was used to perform all statistical analyses.

## 3 Results

### 3.1 Clinical characteristics of CVD

The mean age of the participants was 71.6 ± 9.4 years, with males comprising 73% of the group and an average body mass index (BMI) of 24.2 ± 3.7 kg/m^2^. NT-proBNP was 156 (92.8–464) pg/ml, and laboratory tests including ECG, chest X-P, and echocardiography were within or close to normal levels (Table 1). The number of preserved EF was 23, 7, and 4 cases in non-HF (*n* = 27), HF (*n* = 8), and high-risk HF (*n* = 5), respectively. Beta-blockers were prescribed to 35 of 40 patients (85%) and to 11 of 13 patients with HF (85%) (Supplementary Table S1).

**Table 1:** Clinical characteristics and its relation to heart sound.

	All Patient	S_1_ (dB)	S_2_ (dB)	eS_3_ (dB)	eS_4_ (dB)	ΔS_1_ (dB)	ΔS_2_ (dB)	ΔeS_3_ (dB)	ΔeS_4_ (dB)
	*n* = 40	*r*	*r*	*r*	*r*	*r*	*r*	*r*	*r*
		*p*-value	*p*-value	*p*-value	*p*-value	*p*-value	*p*-value	*p*-value	*p*-value
Age, years	71.6 ± 9.4	0.082	0.008	0.094	0.239	- 0.052	0.041	0.023	0.191
		0.618	0.959	0.566	0.137	0.750	0.803	0.887	0.237
BMI, kg/m^2^	24.2 ± 3.7	−0.379	−0.422	−0.366	−0.543	0.008	0.073	−0.009	−0.116
		0.016	0.007	0.020	<0.001	0.958	0.654	0.956	0.475
NT-proBNP, pg/ml	156 [92.8–464]	−0.217	−0.067	−0.187	−0.270	0.495	0.172	−0.196	−0.034
		0.179	0.681	0.294	0.092	0.001	0.289	0.224	0.835
Cr, mg/dl	1.00 ± 0.21	0.095	0.132	0.029	0.008	0.034	−0.043	−0.179	0.057
		0.562	0.416	0.859	0.960	0.836	0.654	0.269	0.726
eGFR, ml/min/1.73 m^2^	55.1 ± 13.2	−0.040	−0.054	0.025	−0.030	−0.165	0.035	0.028	−0.128
		0.805	0.740	0.880	0.856	0.310	0.831	0.861	0.431
LVEF, %	58.6 ± 8.4	0.084	−0.106	0.031	0.069	−0.200	0.305	−0.051	0.097
		0.606	0.516	0.850	0.674	0.216	0.056	0.755	0.551
E wave, cm/s	69.4 ± 28.9	0.230	0.115	−0.053	−0.196	−0.053	−0.030	−0.252	0.078
		0.073	0.479	0.743	0.225	0.747	0.854	0.117	0.634
A wave, cm/s	68.9 ± 21.8	0.122	0.026	0.220	0.320	−0.299	0.009	−0.031	0.127
		0.501	0.884	0.219	0.069	0.091	0.961	0.864	0.480
E/A	0.99 ± 0.34	0.333	0.247	−0.091	−0.324	0.286	0.116	−0.205	0.007
		0.058	0.165	0.616	0.066	0.106	0.518	0.250	0.971
DcT, msec	234 ± 43.7	0.011	−0.278	0.067	0.097	−0.423	−0.159	0.295	−0.149
		0.957	0.179	0.749	0.643	0.035	0.447	0.152	0.478
E/e′	9.20 ± 3.31	0.041	−0.011	0.067	0.034	−0.096	0.036	−0.113	0.122
		0.804	0.949	0.684	0.836	0.560	0.826	0.492	0.460
TRPG, mmHg	24.2 ± 6.9	−0.020	0.126	−0.168	−0.188	0.211	0.032	−0.255	0.130
		0.908	0.457	0.319	0.266	0.210	0.846	0.127	0.442
QRS, msec	107 ± 20.3	−0.261	−0.015	0.018	0.066	−0.058	−0.206	0.305	0.084
		0.104	0.927	0.912	0.685	0.725	0.202	0.056	0.607
SV1 + RV5, mV	2.42 ± 0.87	−0.113	0.052	0.306	−0.005	−0.117	0.044	−0.147	0.098
		0.488	0.750	0.055	0.976	0.470	0.790	0.362	0.548
CTR, %	48.6 ± 4.2	−0.111	−0.169	−0.178	−0.335	−0.022	−0.051	−0.247	−0.081
		0.495	0.298	0.273	0.035	0.892	0.756	0.124	0.617

Data are presented as means ± SD; BMI, body mass index; CTR, cardiothoracic ratio; Cr, creatinine; DcT, deceleration time; eGFR, estimated glomerular filtration rate; LVEF, left ventricular ejection fraction; NT-proBNP, N-terminal pro-brain natriuretic peptide; TRPG, tricuspid regurgitation peak gradient.

### 3.2 HS quantified in the participants

**Figure 3:**
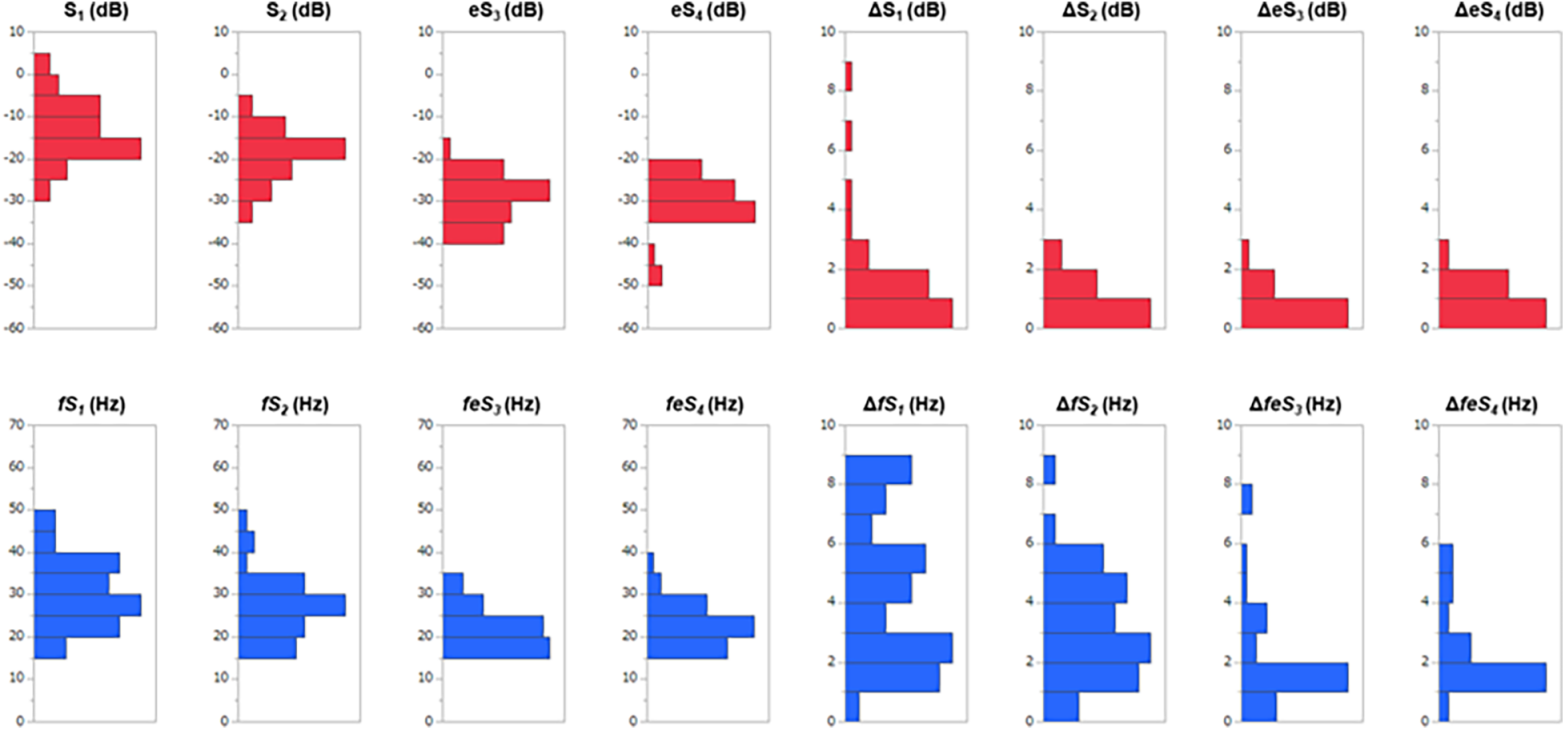
Distributions of sound features and a beat-to-beat difference in 40 CVD patients. S_1_, first heart sound; S_2_, second heart sound; eS_3_, equivalent to third heart sound; eS_4_, equivalent to the fourth heart sound; Δ is the difference between two consecutive beats; the red bars are heart sound intensities; the blue bars are heart sound frequencies.

**Figure 4:**
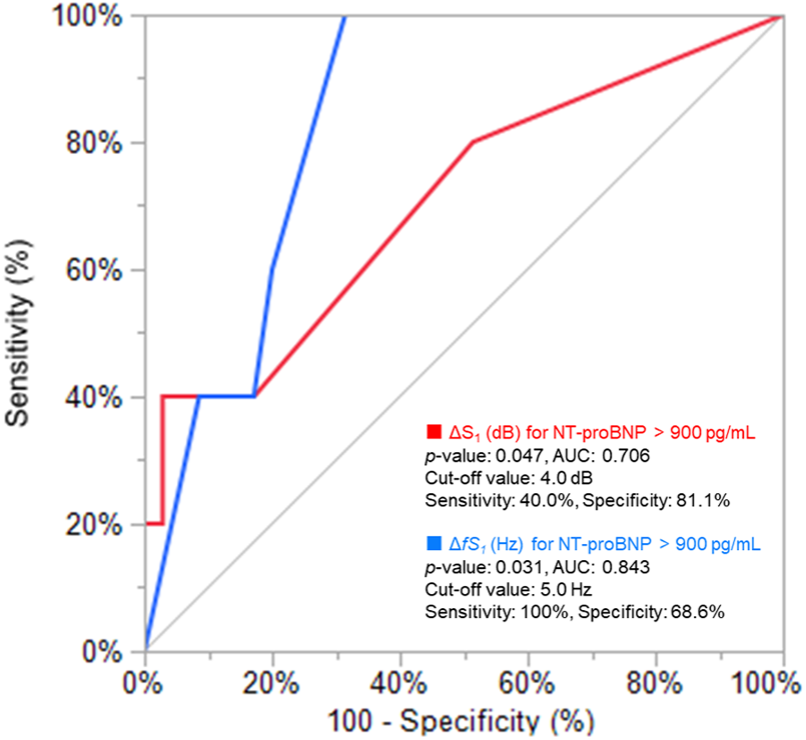
Receiver operating characteristic analysis for CAD patients with high NT-proBNP. AUC, area under the curve; ΔS_1_, acoustic difference (dB) of S_1_ between two consecutive beats; NT-proBNP, N-terminal pro-brain natriuretic peptide.

Distributions of each sound intensity (dB) and frequency (Hz) and a difference between two beats in intensity and frequency of four components are shown in Figure 3. HS intensities of all four components (S_1_–eS_4_) were inversely related to BMI (*p* < 0.02–*p* < 0.001, Table 1, Supplementary Figure S3), and eS_4_ was inversely related to CTR. There was no correlation between HS intensities and NT-proBNP (Supplementary Figure S4). The audio frequency of S_1_ (*fS_1_*) was positively related to BMI and CTR (BMI, *p* = 0.043; CTR, *p* = 0.010; Supplementary Table S2). There was no correlation between HS frequencies and NT-proBNP (Supplementary Figure S5). ΔS_1_ positively correlated with NT-proBNP (*p* = 0.001) and was inversely correlated with deceleration time in echocardiography (*p* = 0.035). On the other hand, Δ*fS_1_* was positively correlated with NT-proBNP and CTR, respectively (NT-proBNP, *p* = 0.033; CTR, *p* = 0.018). As summarized in Table 2, intensities of S_1_, S_2_, eS_3_, and eS_4_ were not different in the presence or absence of HF, while ΔS_1_ (*p* = 0.024) and Δ*fS_1_* (*p* = 0.042) between two consecutive beats were larger in patients with HF than that in patients without HF. These findings were observed where a preceding R–R interval was not different between HF and non-HF. ΔS_1_ (*p* = 0.012) and Δ*fS_1_* (*p* = 0.012) were larger in patients with high-risk HF and were also correlated with AF (*p* = 0.001) or with E/e′ (echocardiographic parameters, *p* = 0.017, Table 3). Multivariate analysis showed that ΔS_1_ was an independent factor from Δ*fS_1_* to predict HF (ΔS_1_, *OR* = 1.659, *p* = 0.044). A group of high-risk HF was correlated significantly with AF (AF, *p* < 0.001, ΔS_1_, *p* = 0.036, and Δ*fS_1_*, *p* = 0.011, Supplementary Table S3). Multivariate analysis showed that Δ*fS_1_* was an independent factor from ΔS_1_ to predict high-risk HF (Δ*fS_1_*, OR = 1.690, *p* = 0.027). AF was a strongly independent factor from HS to predict HF or high-risk HF (Supplementary Table S4). The areas under the curve (AUC) of the ROC for the detection of high-risk HF assessed by ΔS_1_ and Δ*fS_1_* were 0.706 and 0.843 (ΔS_1_, *p* = 0.047; Δ*fS_1_*, *p* = 0.031, Figure 4). The best cutoff values of high-risk HF for ΔS_1_ and Δ*fS_1_* were 4.0 dB and 5.0 Hz, respectively (ΔS_1_, sensitivity 40.0%, specificity 81.1%; Δ*fS_1_*, sensitivity 100%, specificity 68.7%). However, the AUC of the ROC for the detection of HF assessed by ΔS_1_ and Δ*fS_1_* was not significant (Supplementary Figure S6).

**Table 2:** Sound intensity and RR interval in HF and non-HF.

	All	HF	Non-HF	*p*-value	High-risk HF	Non-high-risk HF	*p*-value
	*n* = 40	*n* = 13	*n* = 27	Between HF and none	*n* = 5	*n* = 35	Between high-risk HF and none
S_1_, dB	−13.5 ± 6.51	−14.6 ± 6.9	−13.0 ± 7.16	0.491	−15.2 ± 8.73	−13.3 ± 6.89	0.568
S_2_, dB	−19.8 ± 5.27	−20.2 ± 5.4	−19.6 ± 5.72	0.759	−19.4 ± 5.12	−19.8 ± 5.76	0.881
eS_3_, dB	−30.0 ± 5.34	−30.3 ± 6.6	−30.0 ± 4.93	0.869	−32.6 ± 3.87	−29.7 ± 5.54	0.276
eS_4_, dB	−30.2 ± 5.80	−32.2 ± 6.8	−29.2 ± 5.48	0.134	−32.8 ± 5.40	−29.8 ± 6.13	0.301
ΔS_1_, dB	1.08 ± 1.67	1.92 ± 2.53	0.67 ± 0.83	0.024	2.80 ± 3.27	0.82 ± 1.20	0.012
ΔS_2_, dB	0.51 ± 0.68	0.54 ± 0.66	0.49 ± 0.69	0.844	0.80 ± 0.84	0.47 ± 0.65	0.307
ΔeS_3_, dB	0.33 ± 0.57	0.15 ± 0.37	0.41 ± 0.64	0.193	0.20 ± 0.45	0.34 ± 0.59	0.608
ΔeS_4_, dB	0.48 ± 0.60	0.38 ± 0.51	0.52 ± 0.64	0.515	0.60 ± 0.55	0.46 ± 0.61	0.624
*fS_1_*, Hz	29.2 ± 8.16	29.0 ± 8.79	29.3 ± 7.84	0.904	28.0 ± 8.15	29.4 ± 8.14	0.721
*fS_2_*, Hz	26.5 ± 6.93	28.5 ± 7.61	25.6 ± 6.60	0.228	24.6 ± 5.22	26.8 ± 7.22	0.517
*feS_3_*, Hz	21.4 ± 4.49	20.6 ± 4.61	21.7 ± 4.43	0.462	21.8 ± 2.28	21.3 ± 4.71	0.823
*feS_4_*, Hz	22.1 ± 4.56	21.3 ± 3.73	22.5 ± 4.90	0.134	22.4 ± 3.13	22.1 ± 4.74	0.887
Δ*fS_1_*, Hz	3.87 ± 2.35	5.00 ± 2.19	3.33 ± 2.41	0.042	6.40 ± 1.51	3.51 ± 2.35	0.012
Δ*fS_2_*, Hz	2.75 ± 1.79	3.23 ± 2.31	2.56 ± 1.50	0.273	2.60 ± 1.81	2.80 ± 1.83	0.820
Δ*feS_3_*, Hz	1.63 ± 1.67	1.15 ± 1.21	1.85 ± 1.85	0.225	2.20 ± 1.30	1.54 ± 1.73	0.423
Δ*feS_4_*, Hz	1.75 ± 1.33	1.46 ± 1.19	1.89 ± 1.40	0.350	1.20 ± 0.45	1.83 ± 1.40	0.331
Ambient noise, dB	−39.0 ± 2.54	−39.6 ± 3.72	−39.0 ± 2.41	0.513	−38.4 ± 1.60	−39.3 ± 3.06	0.520
Ambient noise, Hz	53.1 ± 49.1	53.5 ± 50.3	52.9 ± 48.5	0.969	74.6 ± 75.6	50.0 ± 44.1	0.295
RR interval, msec	906 ± 162	890 ± 150	914 ± 171	0.676	845 ± 154	915 ± 164	0.374

Data are presented as means ± SD. Δ is the difference between two beats. HF, heart failure is defined as NT-proBNP > 300 pg/ml. High-risk HF is defined as NT-proBNP > 900 pg/ml; S_1_, first heart sound; S_2_, second heart sound; eS_3_, equivalent to third heart sound; eS_4_, equivalent to the fourth heart sound.

**Table 3:** Univariate/multivariate analysis for HF.

Variable	Univariate analysis for HF			Multivariate analysis for HF		
	OR	95% CI	*p*-value	OR	95% CI	*p*-value
Age, y	1.052	0.971–1.140	0.193	-	-	-
Male, *n* (%)	1.403	0.303–6.494	0.665	-	-	-
BMI, kg/m^2^	0.905	0.847–1.331	0.598	-	-	-
AF, *n* (%)	22.28	2.290–216.9	0.001	-	-	-
IHD, *n* (%)	0.428	0.111–1.657	0.217	-	-	-
Cr, mg/dl	9.046	0.360–27.16	0.168	-	-	-
eGFR, ml/min/1.73 m^2^	0.956	0.897–1.019	0.128	-	-	-
LVEF, %	1.031	0.947–1.121	0.474	-	-	-
E wave, cm/s	1.018	0.993–1.042	0.144	-	-	-
DcT, msec	0.988	0.967–1.010	0.250	-	-	-
E/e′	1.324	1.006–1.742	0.017	-	-	-
TRPG, mmHg	1.023	0.925–1.133	0.654	-	-	-
S_1_, dB	0.965	0.874–1.065	0.475	-	-	-
S_2_, dB	0.981	0.870–1.106	0.752	-	-	-
eS_3_, dB	0.989	0.874–1.119	0.865	-	-	-
eS_4_, dB	0.918	0.815–1.033	0.133	-	-	-
ΔS_1_, dB	1.683	0.966–2.932	0.024	1.659	0.943–2.918	0.044
ΔS_2_, dB	1.107	0.414–2.957	0.839	-	-	-
ΔeS_3_, dB	0.369	0.079–1.720	0.156	-	-	-
ΔeS_4_, dB	0.670	0.206–2.174	0.496	-	-	-
*fS_1_*, Hz	0.995	0.925–1.093	0.901	-	-	-
*fS_2_*, Hz	1.062	0.854–1.038	0.220	-	-	-
*feS_3_*, Hz	0.941	0.907–1.246	0.443	-	-	-
*feS_4_*, Hz	0.939	0.911–1.244	0.417	-	-	-
Δ*fS_1_*, Hz	1.344	1.000–1.805	0.040	1.319	0.964–1.805	0.074
Δ*fS_2_*, Hz	1.236	0.555–1.179	0.264	-	-	-
Δ*feS_3_*, Hz	0.723	0.806–2.373	0.182	-	-	-
Δ*feS_4_*, Hz	0.758	0.741–2.345	0.320	-	-	-

AF, atrial fibrillation; BMI, body mass index; CI, confidence interval; CTR, cardiothoracic ratio; Cr, creatinine; DcT, deceleration time; eGFR, estimated glomerular filtration rate; HF, heart failure is defined as NT-proBNP > 300 pg/ml; IHD, ischemic heart disease, LVEF, left ventricular ejection fraction; NT-proBNP, N-terminal pro-brain natriuretic peptide; OR, odd ratio; S_1_, first heart sound; S_2_, second heart sound; eS_3_, equivalent to third heart sound; eS_4_, equivalent to the fourth heart sound, TRPG, tricuspid regurgitation peak gradient.

### 3.3 Detections of HF by HS

**Figure 5:**
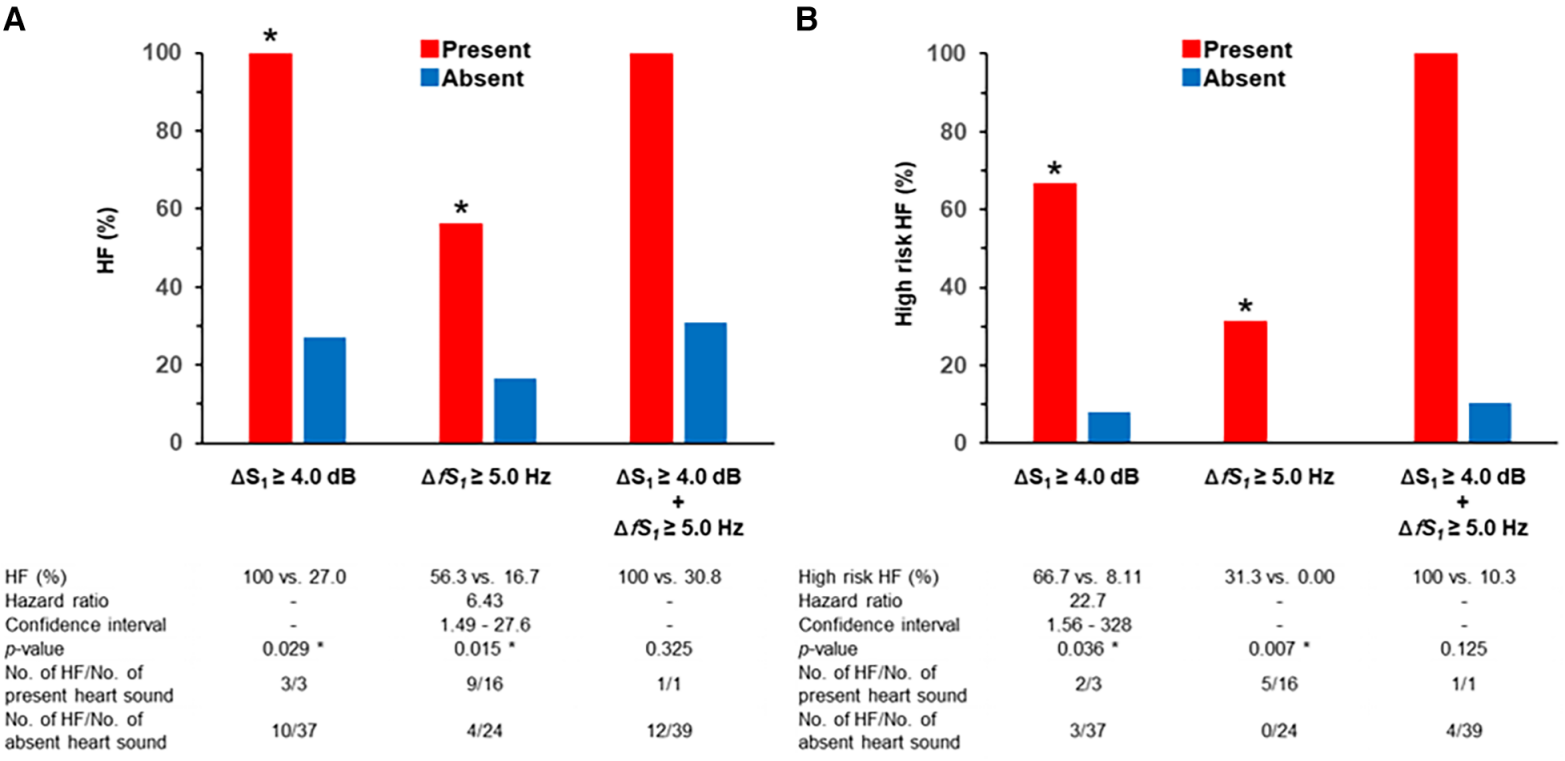
Incidence of HF and heart sound alterations. * is *p* < 0.050; Δ is the difference between two beats; HF, heart failure; S_1_, first heart sound. (**A**) HF is defined as NT-proBNP > 300 pg/ml. (**B**) High-risk HF is defined as NT-proBNP > 900 pg/ml.

The incidence of HF was significantly higher in patients with ΔS_1_ ≥ 4.0 dB than those with ΔS_1_ < 4.0 dB (100 vs., 27.0%, *p* = 0.029). The incidence of HF was also significantly higher in patients with Δ*fS_1_* ≥ 5.0 Hz than that of those without (56.3 vs. 16.7%, *p* = 0.015). Only one participant met both ΔS_1_ ≥ 4.0 dB and Δ*fS_1_* ≥ 5.0 Hz, and the incidence of HF tended to be high although the statistical difference was not performed (Figures 5A,B). The incidence of high-risk HF was significantly higher in patients with ΔS_1_ ≥ 4.0 dB than those without (66.7 vs. 8.1%, *p* = 0.036). The incidence of HF was also significantly higher in patients with Δ*fS_1_* ≥ 5.0 Hz than those without (31.3 vs. 0.00%, *p* = 0.007). Among the three groups according to the NT-proBNP, ΔS_1_ and Δ*fS_1_* in patients with NT-proBNP over 900 pg/ml were significantly larger than those in patients with NT-proBNP below 300 pg/ml (ΔS_1_, *p* = 0.004; Δ*fS_1_*, *p* = 0.011, Supplementary Figure S7) although statistical significance was not observed between the other groups.

## 4 Discussion

The main findings of this study were (1) quantitative sound analysis on acoustic measures through digital data acquisition and processing is necessary to overcome the auscultatory proficiency related to the subjectivity of the examiner, (2) the intensity of all components of HS was inversely related to the level of BMI, and (3) S_1_ alterations between two consecutive beats in intensity and frequency at the peak power of S_1_ spectrum were associated with levels of NT-proBNP.

### 4.1 Quantifying heart sound

There are many aspects in quantifying the clinical features of HS. For describing physical signs, physicians documented semiquantitatively by loudness, pitch, and tone with verbal expressions through the perception of the physician (23). A phonocardiogram is powerful in determining the timing of sound occurrence in relation to ECG; however, how to compare patients' variations remains unexplored. Reddy et al. (24) applied a calibrated pressure mechanocardiograph to measure sound strength, but pitch or tone was not measured. To overcome subjective differences in audibility or visual recognition of phonocardiogram, we introduced a digital acquisition and processing of HS.

Physical appropriateness of our auscultatory device, handheld tri-antler chest piece (BMP-LD3) was supported firstly by assessing the influence of BMI on the HS and secondly by recordings of tiny and barely audible vibrations around the timings of S_3_ and S_4_. It is known that the acoustic intensity of HS declines by the distance between the heart and the thorax surface, the volumes of the lung (25), and the amount of other sound absorbers such as connective, adipose, and muscle tissues (26). Luo et al. demonstrated a significant intensity drop in HS recordings in cases with BMI > 30 kg/m^2^, when physicians applied smartphones in the hospital for auscultation (27). In the present study, we observed an inverse relation between the intensity of HS and the levels of BMI (BMI between 16.2 and 33.0 kg/m^2^, *n* = 40, aged from 46 to 87 years). In addition, HS intensity in aged patients was weak (24), and stored data were augmented in the digital analysis in the present study. Accordingly, we understand that the present device has sufficient resolution to quantify HS acoustically.

### 4.2 Sensitive biomarkers in heart failure

HS have been accepted as a cardiac biomarker in patients with HF (28). To detect occurrences of HF, wearable devices such as a smartwatch or a smartphone (29, 30) are available along with an implanted long-term (31) and continuous monitoring device to observe ECG and cardiac sounds for the management of HF (32). Artificial intelligence analysis of ECG and phonocardiogram was challenged to predict the occurrence of HF (33–35). These studies focused on the presence of diastolic HS such as S_3_ or S_4_; however, S_3_ or S_4_ was not evident in the present participants; they were all ambulatory and did not exhibit overt HF upon visiting our outpatient clinic, and 13 of them (32.5%) had HF defined by NT-proBNP level ≥ 300 pg/ml.

Although HF in the present participants was hemodynamically and clinically mild and 85% of HF participants were under the control of beta-blockades, the quantitative sound analysis revealed that (1) a beat-to-beat alteration of S_1_ intensity (dB) and its audio frequency (Hz) was significantly associated with the level of NT-proBNP and (2) the size of CTR correlated negatively with eS_4_ intensity (*p* = 0.035) and positively with S_1_ audio frequency (*p* = 0.010) (Table 1). In addition, highly significant differences in ΔS_1_ intensity and frequency were noted between HF and non-HF. Since such peculiar findings were not observed in S_2_, eS_3_, or eS_4_, these findings may suggest a latent cardio-mechanistic derangement in HF.

### 4.3 Mechanisms of S_1_ beat-to-beat alteration

In previous case reports in patients with severe HF, S_1_ was documented to be soft and muffled because of congestive blood flow and poor heart pumping (13, 14). S_1_ appears during the isovolumic contraction when the left ventricular (LV) dP/dt reaches its peak level. A tight relationship between the maximum positive LVdP/dt and the amplitude of S_1_ was repeatedly demonstrated in animal studies (36, 37). The level of peak LV dP/dt has been accepted as a standard index of contractility (38). Then, the relationship between S_1_ sound characteristics and LV contractility may be described by a simple first-order system. However, a highly accurate pressure measurement was not realistic for participants in the outpatient clinic. To prove how well a simple first-order system fits to the relationship between S_1_ alteration and LV contractility remains one of the important future studies.

In the Framingham Heart Study, AF occurs in more than half of individuals with HF, and HF occurs in more than one-third of individuals with AF (39). HF and AF also frequently coexist. AF precedes and follows HF with both preserved and reduced EF (40). A previous study also reported that AF is highly predictive of underlying HFpEF (41). H_2_FPEF score was calculated by clinical variables including heavy, hypertensive, AF, pulmonary hypertension, and elder and filling pressure. Subsequently, AF accounts for 3 of 9 points in the H_2_FPEF score (42). HFA-PEFF score was also calculated by functional, morphological, and biomarker domains. AF is a main factor in the initial evaluation of the HFA-PEFF diagnostic algorithm (43), although both scores have good sensitivity and specificity to detect HFpEF (44). In the present study, AF was noted in 11 of 13 participants with HF and they were classified as HFpEF. Equal RR interval in these participants may indicate dysfunction of the autonomic nervous system (45). Accordingly, a beat-to-beat S_1_ alteration may represent mechanisms similar to pulsus alternans (46).

### 4.4 Sound alteration of non-audible level in heart failure

An audible range of humans is at least ≥0 dB at the audio frequencies between 20 Hz and 20 kHz (47). In human ears, the audible minimum change of sound pressure is at least ≥2 dB (23) although the audible threshold worsens according to age. In the present study, the detected sound signal was augmented electrically, which enabled us to recognize slight changes in HS on PCG recordings. In the present augmented signals, absolute differences in S_1_ intensity and its peak audio frequency between consecutive beats were 4.0 dB and 5.0 Hz, and such tiny changes were assessable due to the digital quantification of sound signals.

### 4.5 Limitations

There are several limitations: (1) the number of participants was limited in the present study; however, subjects from the real world of daily clinical activities provide the present research eliminating biases in the selection of participants, (2) participants with the absence of CVD were not included, (3) we did not study the difference between physician's audibility and digital acquisition of HS, and (4) since the present study is based on a small number of participants, the contribution of this single-center exploratory study remains a hypothesis generation for future further studies.

## Conclusions

The practical role of auscultation has been discussed a lot, before and following the 200th anniversary of the stethoscope (48). Although S_3_ and S_4_ in HF have been defined by auscultatory recognition, their sensitivity and specificity in the physical examination are limited by the physician's proficiency and audibility (47, 49). The present findings on HF-associated S_1_ beat-to-beat alteration in sound characteristics propose a strong need for a highly sensitive stethoscope and its quantitative assessment on HS below audible level.

## Data Availability

The raw data supporting the conclusions of this article will be made available by the authors, without undue reservation.
