# Supplementary material for: Beat-to-beat alterations of acoustic intensity and frequency at the maximum power of heart sounds are associated with NT-proBNP levels
Source: Front Cardiovasc Med. 2024 Apr 2;11:1372543. doi: 10.3389/fcvm.2024.1372543 (PMC11018890; doi:10.3389/fcvm.2024.1372543)
Supplement: Supplementary file 1 [file Datasheet1.docx]

**Beat to beat alterations of acoustic intensity and frequency at the maximum power of heart sounds are associated with NT-proBNP levels**

Kazuhiro Fujiyoshi, MD, PhD^1^, Minako Yamaoka-Tojo, MD, PhD^2^, Kanako Fujiyoshi, CLT^2^, Takumi Komatsu, BS^3^, Jun Oikawa, MD, PhD^4^, Kunio Kashino, PhD^5^, Hitonobu Tomoike, MD, PhD^5^, Junya Ako, MD, PhD^1^

1. Department of Cardiovascular Medicine, Kitasato University School of Medicine, Sagamihara, Japan
2. Department of Rehabilitation, Kitasato University School of Allied Health Sciences, Sagamihara, Japan.
3. Department of Functional Restoration Science, Kitasato University Graduate School of Medical Sciences, Sagamihara, Japan.
4. Department of Kitasato Clinical Research Center, Kitasato University School of Medicine, Sagamihara, Japan
5. Bio-Medical Informatics Research Center, NTT Basic Research Laboratories, Atsugi, Japan

Supplementary tables S1-4

Supplementary figures S1-7

Table S1. Clinical characteristics in HF and non-HF

|  | **All**  **n = 40** | **HF**  **n = 13** | **Non-HF**  **n = 27** | ***p*-value**  **between HF**  **and Non-HF** | **High risk HF**  **n = 5** | **Non-high risk HF**  **n = 35** | ***p*-value**  **between high risk HF**  **and Non-high risk HF** |
| --- | --- | --- | --- | --- | --- | --- | --- |
| Male, n (%) | 29 (73) | 10 (77) | 19 (70) | 0..664 | 4 (80) | 25 (71) | 0.688 |
| Type of CVD |  |  |  |  |  |  |  |
| IHD, n (%) | 24 (60) | 6 (46) | 18 (67) | 0.215 | 1 (20) | 23 (65) | 0.051 |
| AF, n (%) | 7 (18) | 6 (46) | 1 (4) | < 0.001 | 4 (80) | 3 (9) | < 0.001 |
| AS, n (%) | 11 (28) | 5 (38) | 6 (22) | 0.281 | 2 (40) | 9 (26) | 0.503 |
| AR, n (%) | 7 (18) | 4 (31) | 3 (11) | 0.125 | 2 (40) | 5 (14) | 0.157 |
| MR, n (%) | 15 (38) | 5 (38) | 10 (37) | 0.930 | 4 (80) | 11 (31) | 0.036 |
| TR, n (%) | 20 (50) | 10 (76) | 10 (37) | 0.018 | 5 (100) | 15 (43) | 0.017 |
| Type of HF |  |  |  |  |  |  |  |
| HFpEF | 22 (55) | 9 (69) | 13 (48) | 0.209 | 3 (60) | 19 (54) | 0.810 |
| HFmrEF | 12 (30) | 2 (15) | 11 (84) | 0.162 | 1 (20) | 11 (31) | 0.602 |
| HFrEF | 6 (15) | 2 (15) | 4 (15) | 0.962 | 1 (20) | 5 (14) | 0.738 |
| Medications |  |  |  |  |  |  |  |
| ACEI/ARB, n (%) | 28 (70) | 9 (69) | 19 (70) | 0.941 | 3 (60) | 25 (71) | 0.602 |
| ARNI, n (%) | 0 (0) | 0 (0) | 0 (0) | - | 0 (0) | 0 (0) | - |
| Beta-blocker, n (%) | 35 (88) | 11 (84) | 24 (89) | 0.702 | 5 (100) | 30 (86) | 0.366 |
| Diuretics, n (%) | 8 (20) | 5 (38) | 3 (11) | 0.043 | 3 (60) | 5 (14) | 0.017 |
| SGLT2i, n (%) | 2 (5) | 1 (8) | 1 (4) | 0.588 | 0 (0) | 2 (6) | 0.583 |
| Statin, n (%) | 27 (68) | 7 (54) | 20 (74) | 0.201 | 1 (20) | 26 (74) | 0.015 |

Data are presented as n (%). HF, heart failure is defined as NT-proBNP > 300 pg/mL; High risk HF is defined as NT-proBNP > 900 pg/mL; ACEI, angiotensin converting enzyme; AF, atrial fibrillation; AR, aortic valve regurgitation; ARB, angiotensin II receptor blocker; ARNI, angiotensin receptor neprilysin inhibitor; AS, aortic valve stenosis; CVD, cardiovascular disease; HF, heart failure; HFpEF, HF preserved ejection fraction; HFmrEF, HF mild reduced ejection fraction; HFrEF, HF reduced ejection fraction; IHD, ischemic heart disease; MR, mitral valve regurgitation; SGLT2i, sodium glucose cotransporter inhibitor; TR, tricuspid valve regurgitation.

Table S2. Clinical characteristics and it’s relation to heart sound

|  | ***fS_1_* (Hz)** | ***fS_2_* (Hz)** | ***feS_3_* (Hz)** | ***feS_4_* (Hz)** |  | **Δ*fS_1_* (Hz)** | **Δ*fS_2_* (Hz)** | **Δ*feS_3_* (Hz)** | **Δ*feS_4_* (Hz)** |
| --- | --- | --- | --- | --- | --- | --- | --- | --- | --- |
|  | ***r***  ***p*-value** | ***r***  ***p*-value** | ***r***  ***p*-value** | ***r***  ***p*-value** |  | ***r***  ***p*-value** | ***r***  ***p*-value** | ***r***  ***p*-value** | ***r***  ***p*-value** |
| Age, y | - 0.096  0.554 | 0.116  0.478 | - 0.057  0.725 | 0.118  0.469 |  | 0.060  0.713 | - 0.049  0.765 | - 0.085  0.603 | - 0.044  0.786 |
| BMI, kg/m^2^ | 0.320  0.043 | - 0.348  0.078 | 0.177  0.264 | - 0.085  0.600 |  | 0.116  0.476 | 0.197  0.223 | 0.016  0.923 | 0.132  0.416 |
| NT-proBNP, pg/mL | - 0.070  0.668 | - 0.050  0.759 | - 0.008  0.962 | - 0.070  0.668 |  | 0.458  0.003 | 0.011  0.945 | 0.043  0.792 | - 0.133  0.412 |
| Cr, mg/dL | - 0.019  0.906 | 0.166  0.307 | 0.070  0.666 | 0.006  0.970 |  | 0.166  0.306 | - 0.039  0.813 | - 0.070  0.668 | 0.033  0.841 |
| eGFR, mL/min/1.73m^2^ | - 0.083  0.612 | - 0.286  0.074 | - 0.059  0.717 | - 0.071  0.665 |  | - 0.167  0.303 | - 0.043  0.792 | - 0.065  0.692 | - 0.167  0.302 |
| LVEF, % | 0.199  0.218 | 0.036  0.825 | - 0.005  0.976 | - 0.047  0.774 |  | 0.033  0.838 | - 0.024  0.885 | 0.032  0.846 | 0.149  0.358 |
| E wave, cm/s | 0.159  0.327 | 0.009  0.958 | - 0.081  0.619 | - 0.070  0.668 |  | 0.019  0.906 | 0.027  0.869 | - 0.198  0.220 | 0.103  0.528 |
| A wave, cm/s | - 0.003  0.988 | 0.193  0.282 | 0.135  0.455 | - 0.177  0.325 |  | - 0.250  0.075 | 0.089  0.622 | - 0.006  0.997 | 0.184  0.305 |
| E/A | - 0.126  0.486 | - 0.255  0.152 | - 0.232  0.194 | - 0.006  0.974 |  | 0.068  0.708 | - 0.285  0.107 | - 0.242  0.174 | - 0.066  0.713 |
| DcT, msec | 0.348  0.088 | 0.228  0.273 | - 0.032  0.878 | 0.068  0.746 |  | 0.189  0.365 | 0.344  0.086 | - 0.007  0.971 | 0.051  0.807 |
| E/e’ | - 0.038  0.818 | 0.191  0.245 | - 0.282  0.082 | - 0.124  0.452 |  | - 0.119  0.471 | 0.055  0.738 | -0.221  0.177 | - 0.191  0.244 |
| TRPG, mmHg | 0.032  0.849 | 0.005  0.977 | - 0.047  0.781 | - 0.213  0.206 |  | 0.276  0.099 | - 0.104  0.540 | - 0.038  0.823 | - 0.130  0.443 |
| QRS, msec | - 0.307  0.054 | - 0.026  0.876 | 0.120  0.459 | 0.068  0.675 |  | - 0.011  0.947 | 0.002  0.986 | - 0.010  0.952 | - 0.001  0.994 |
| SV1+RV5, mV | - 0.002  0.992 | 0.142  0.384 | 0.109  0.501 | 0.099  0.543 |  | - 0.093  0.566 | 0.269  0.093 | - 0.669  0.669 | 0.043  0.789 |
| CTR, % | 0.404  0.010 | 0.082  0.613 | 0.109  0.501 | 0.003  0.985 |  | 0.372  0.018 | 0.103  0.527 | - 0.077  0.638 | 0.094  0.564 |

Data are presented as means ± SD; BMI, body mass index; CTR, cardio-thoracic ratio; Cr, creatinine; DcT, deceleration time; eGFR, estimated glomerular filtration rate; LVEF, left ventricular ejection fraction; NT-proBNP, N-terminal pro-brain natriuretic peptide; TRPG, tricuspid regurgitation peak gradient.

Table S3. Univariate/Multivariate analysis for high risk HF

| **Variable** | **Univariate analysis for high risk HF** | | |  | **Multivariate analysis for high risk HF** | | |
| --- | --- | --- | --- | --- | --- | --- | --- |
|  | **OR** | **95% CI** | ***p*-value** |  | **OR** | **95% CI** | ***p*-value** |
| Age, y | 1.084 | 0.949 - 1.240 | 0.193 |  | - | - | - |
| Male, n (%) | 1.600 | 0.159 - 16.13 | 0.690 |  | - | - | - |
| BMI, kg/m^2^ | 0.917 | 0.701 - 1.200 | 0.523 |  | - | - | - |
| AF, n (%) | 42.67 | 3.536 - 514.8 | < 0.001 |  | - | - | - |
| IHD, n (%) | 0.130 | 0.013 - 1.301 | 0.083 |  | - | - | - |
| Cr, mg/dL | 10.04 | 0.210 - 48.05 | 0.256 |  |  |  |  |
| eGFR, mL/min/1.73m^2^ | 0.946 | 0.859 - 1.042 | 0.217 |  |  |  |  |
| LVEF, % | 1.005 | 0.895 - 1.127 | 0.937 |  | - | - | - |
| E wave, cm/s | 1.014 | 0.984 - 1.045 | 0.370 |  | - | - | - |
| DcT, msec | 0.986 | 0.954 - 1.020 | 0.411 |  | - | - | - |
| E/e’ | 1.219 | 0.962 - 1.546 | 0.105 |  | - | - | - |
| TRPG, mmHg | 1.150 | 0.996 - 1.327 | 0.045 |  | - | - | - |
| S_1_, dB | 0.958 | 0.831 - 1.105 | 0.551 |  | - | - | - |
| S_2_, dB | 1.014 | 0.854 - 1.203 | 0.876 |  | - | - | - |
| eS_3_, dB | 0.904 | 0.754- 1.084 | 0.264 |  | - | - | - |
| eS_4_, dB | 0.932 | 0.816 - 1.067 | 0.325 |  | - | - | - |
| ΔS_1_, dB | 1.623 | 1.007 - 2.617 | 0.036 |  | 1.651 | 0.851 - 3.203 | 0.090 |
| ΔS_2_, dB | 1.948 | 0.542 - 6.999 | 0.316 |  | - | - | - |
| ΔeS_3_, dB | 0.582 | 0.076 - 4.464 | 0.575 |  | - | - | - |
| ΔeS_4_, dB | 1.470 | 0.327 - 6.600 | 0.621 |  | - | - | - |
| *fS_1_*, Hz | 0.978 | 0.905 - 1.156 | 0.710 |  | - | - | - |
| *fS_2_*, Hz | 0.950 | 0.904 - 1.225 | 0.491 |  | - | - | - |
| *feS_3_*, Hz | 1.024 | 0.794 - 1.200 | 0.820 |  | - | - | - |
| *feS_4_*, Hz | 1.016 | 0.801 - 1.210 | 0.884 |  | - | - | - |
| Δ*fS_1_*, Hz | 1.758 | 1.051 - 2.937 | 0.011 |  | 1.690 | 0.987 - 2.893 | 0.027 |
| Δ*fS_2_*, Hz | 0.937 | 0.621 - 1.832 | 0.813 |  | - | - | - |
| Δ*feS_3_*, Hz | 1.214 | 0.512 - 1.322 | 0.442 |  | - | - | - |
| Δ*feS_4_*, Hz | 0.584 | 0.559 - 5.244 | 0.262 |  | - | - | - |

AF, atrial fibrillation; BMI, body mass index; CI, confidence interval; CTR, cardio-thoracic ratio; Cr, creatinine; DcT, deceleration time; eGFR, estimated glomerular filtration rate; HF, heart failure is defined as NT-proBNP > 300 pg/mL; IHD, ischemic heart disease, LVEF, left ventricular ejection fraction; NT-proBNP, N-terminal pro-brain natriuretic peptide; OR, odd ratio; S_1_, first heart sound; S_2_, second heart sound; eS_3_, equivalent to third heart sound; eS_4_, equivalent to forth heart sound, TRPG, tricuspid regurgitation peak gradient.

Table S4. Multivariate analysis for HF and high risk HF

| **Variable** | **Multivariate analysis**  **for HF** | | |  | **Multivariate analysis**  **for HF** | | |  | **Multivariate analysis**  **for high risk HF** | | |  | **Multivariate analysis**  **for high risk HF** | | |
| --- | --- | --- | --- | --- | --- | --- | --- | --- | --- | --- | --- | --- | --- | --- | --- |
|  | **OR** | **95% CI** | ***p*-value** |  | **OR** | **95% CI** | ***p*-value** |  | **OR** | **95% CI** | ***p*-value** |  | **OR** | **95% CI** | ***p*-value** |
| AF, n (%) | 15.31 | 1.219 - 192.3 | 0.035 |  | 17.31 | 1.699 - 176.5 | 0.016 |  | 36.90 | 2.224 - 612.3 | 0.012 |  | 37.74 | 2.215 - 576.7 | 0.012 |
| ΔS_1_, dB | 4.507 | 0.018 - 113.5 | 0.579 |  | - | - | - |  | 1.061 | 0.618 - 1.819 | 0.829 |  | - | - | - |
| Δ*fS_1_*, Hz | - | - | - |  | 5.512 | 0.397 - 76.60 | 0.201 |  | - | - | - |  | 1.799 | 0.845 - 3.827 | 0.061 |

AF, atrial fibrillation; HF, heart failure is defined as NT-proBNP > 300 pg/mL; NT-proBNP, N-terminal pro-brain natriuretic peptide; OR, odd ratio; S_1_, first heart sound.

**Figure S1. Heart sound collection**


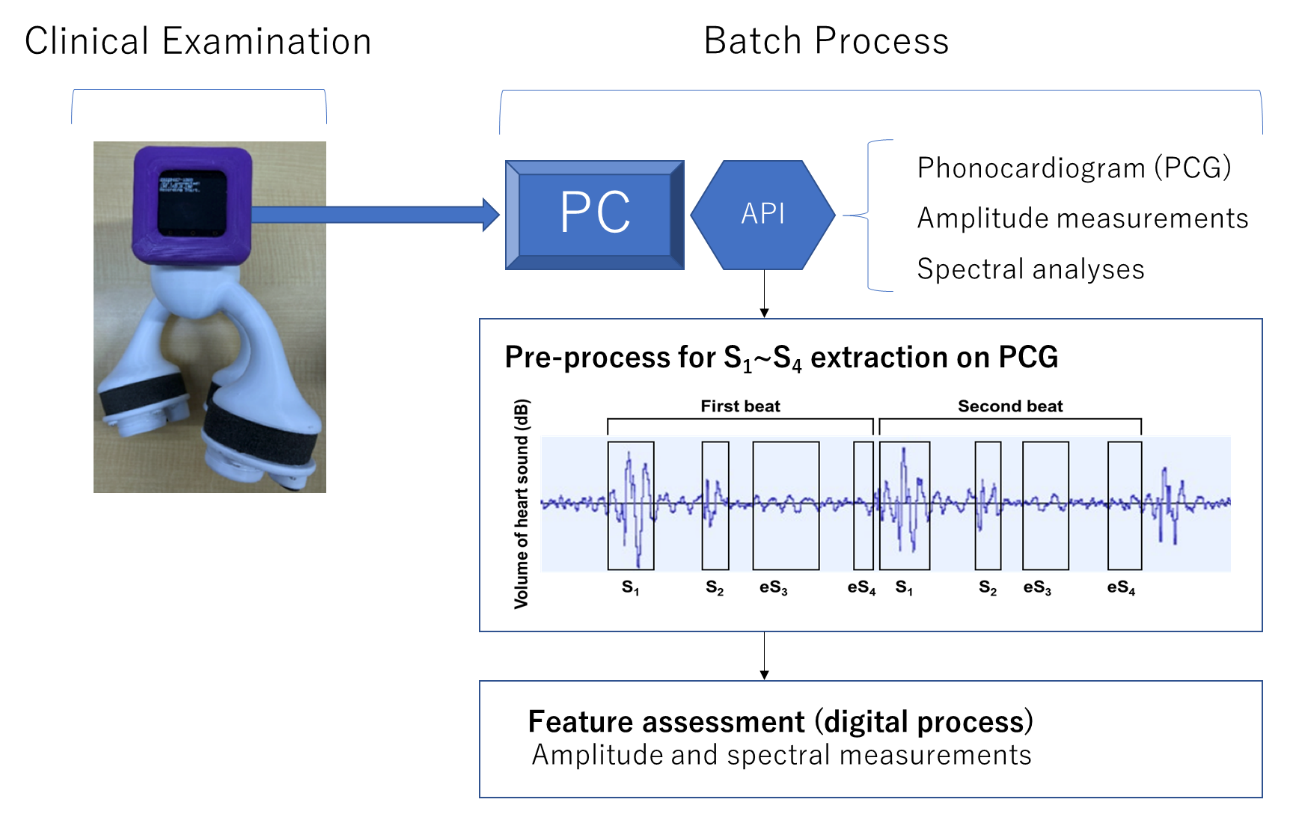


API, application programming interface; S_1_, first heart sound; S_2_, second heart sound; eS_3_, equivalent to third heart sound; eS_4_, equivalent to forth heart sound; PC, personal computer; PCG, phonocardiogram.

**Figure S2. Definitions of ECG wave**


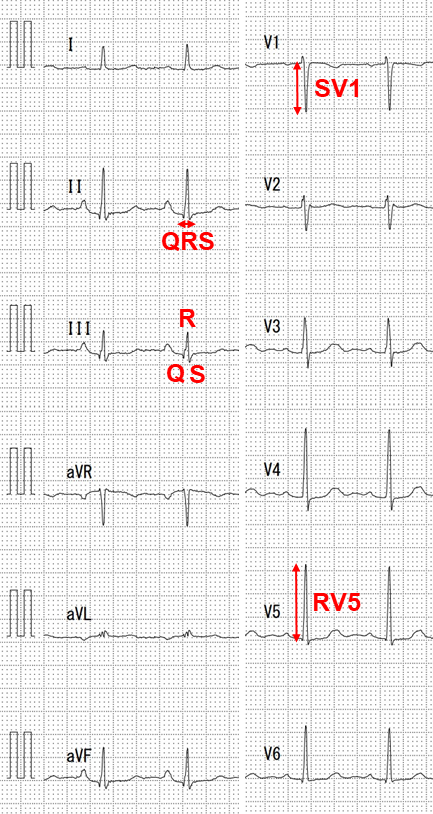


**Figure S3. Correlations between heart sounds and BMI**


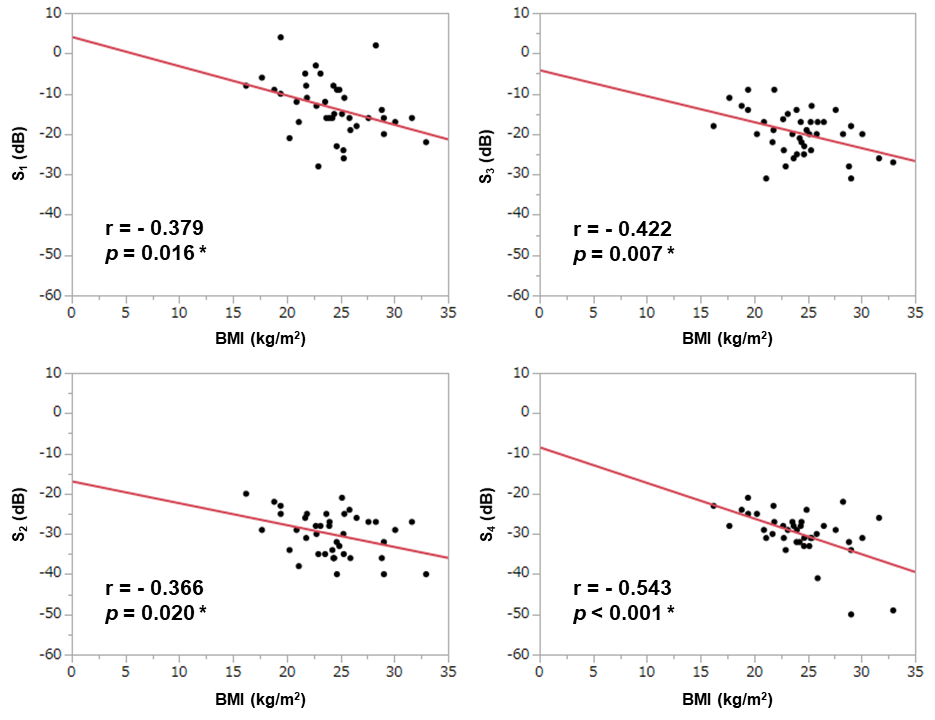


*, *p* <0.050; BMI, body mass index; S_1_, first heart sound; S_2_, second heart sound; eS_3_, equivalent to third heart sound; eS4, equivalent to forth heart sound.

**Figure S4. Correlations between heart sounds intensities and NT-proBNP**


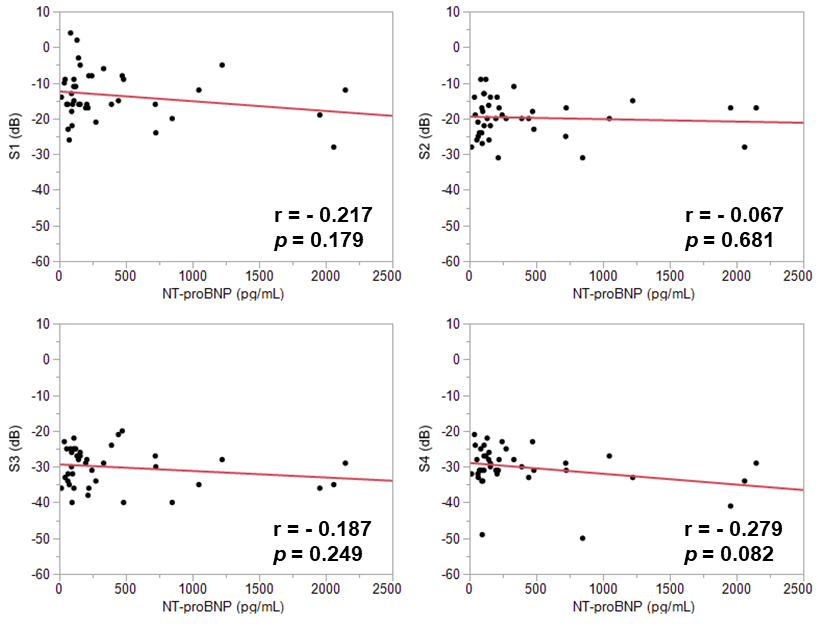


NTproBNP, N-terminal pro-brain natriuretic peptide; S_1_, first heart sound; S_2_, second heart sound; eS_3_, equivalent to third heart sound; eS4, equivalent to forth heart sound.

**Figure S5. Correlations between heart sounds frequencies and NT-proBNP**


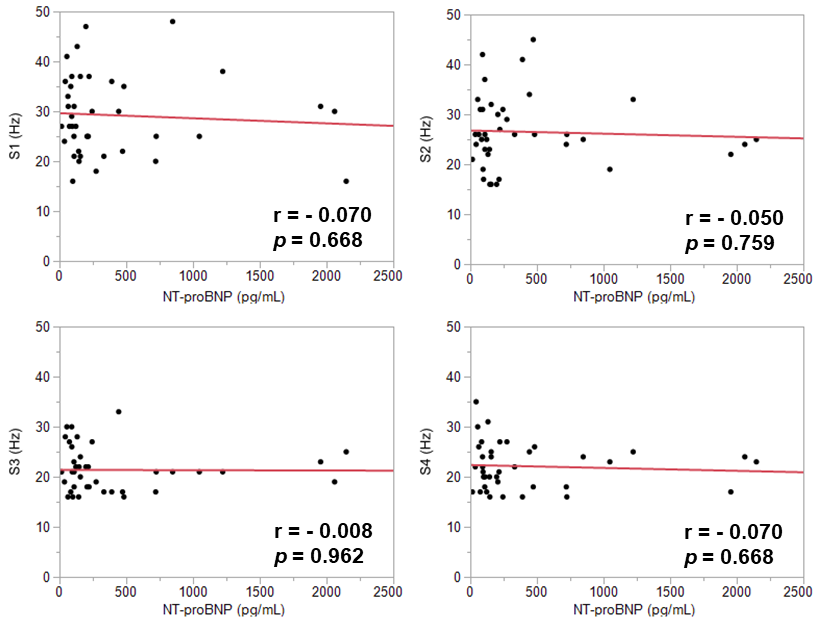


NT-proBNP, N-terminal pro-brain natriuretic peptide; S_1_, first heart sound; S_2_, second heart sound; eS_3_, equivalent to third heart sound; eS4, equivalent to forth heart sound.

**Figure S6. Receiver operating characteristic analysis for heart failure**

**
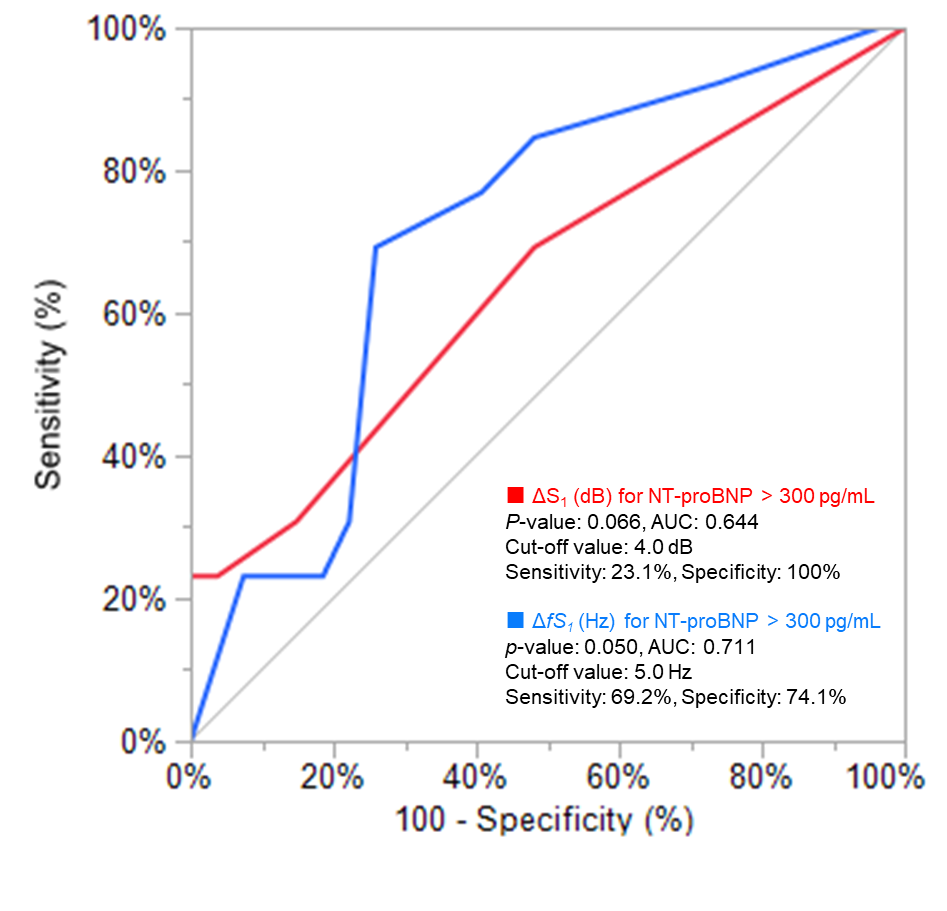
**

AUC, area under curve; ΔS_1_, absolute value of difference between first heart sound continuous 2 beat; NT-proBNP, N-terminal pro-brain natriuretic peptide.

**Figure S7. ΔS_1_ intensities according to NT-proBNP**


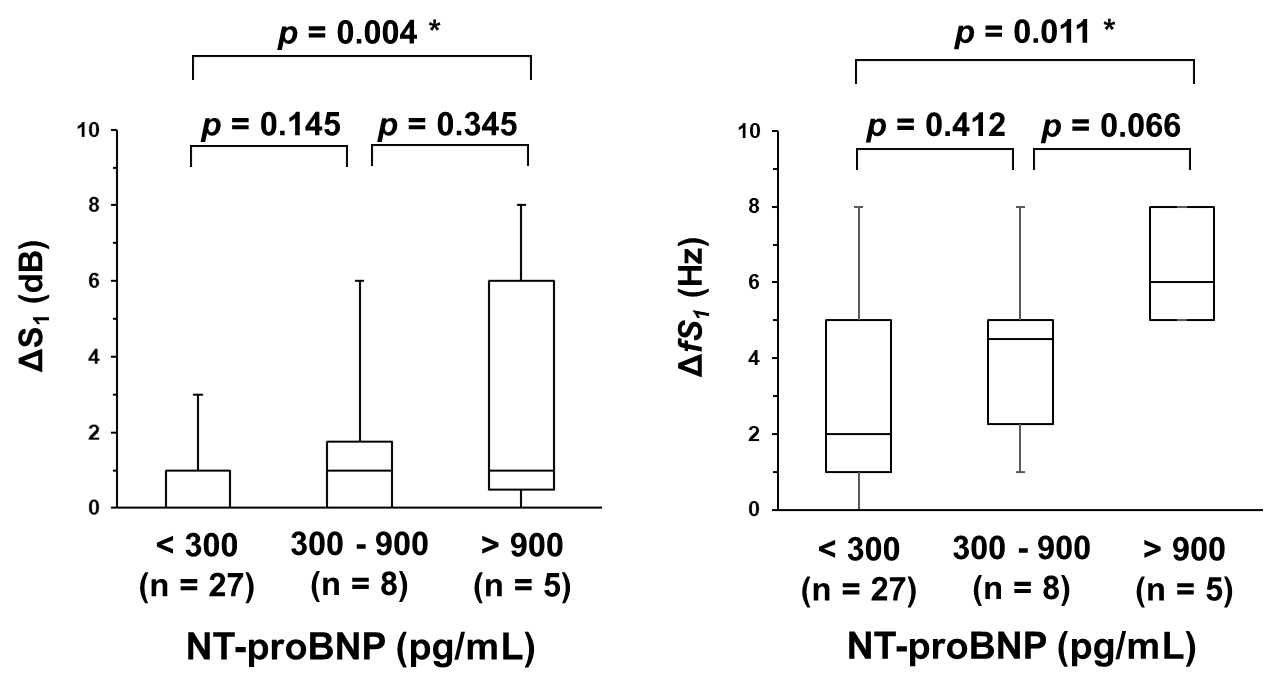


*, *p* < 0.050; NT-proBNP, N-terminal pro-brain natriuretic peptide; ΔS_1_, absolute value of difference between first heart sound continuous 2 beat
